# Research on Ship Replenishment Path Planning Based on the Modified Whale Optimization Algorithm

**DOI:** 10.3390/biomimetics10030179

**Published:** 2025-03-13

**Authors:** Qinghua Chen, Gang Yao, Lin Yang, Tangying Liu, Jin Sun, Shuxiang Cai

**Affiliations:** 1 Navy Aviation University, Yantai 264001, China; ytcqh205@126.com (Q.C.); sj15071064649@outlook.com (J.S.); 2 School of Electromechanical and Automotive Engineering, Yantai University, Yantai 264005, China; lty@ytu.edu.cn (T.L.); caisx8411@ytu.edu.cn (S.C.)

**Keywords:** ship replenishment path planning, modified whale optimization algorithm, nearest neighbor search, cross operation, destroy and repair operators, variable neighborhood search

## Abstract

Ship replenishment path planning has always been a critical concern for researchers in the field of security. This study proposes a modified whale optimization algorithm (MWOA) to address single-task ship replenishment path planning problems. To ensure high-quality initial solutions and maintain population diversity, a hybrid approach combining the nearest neighbor search with random search is employed for initial population generation. Additionally, crossover operations and destroy and repair operators are integrated to update the whale’s position, significantly enhancing the algorithm’s search efficiency and optimization performance. Furthermore, variable neighborhood search is utilized for local optimization to refine the solutions. The proposed MWOA has been tested against several algorithms, including the original whale optimization algorithm, genetic algorithm, ant colony optimization, hybrid particle swarm optimization, and simulated annealing, using traveling salesman problems as benchmarks. Results demonstrate that MWOA outperforms these algorithms in both solution quality and stability. Moreover, when applied to ship replenishment path planning problems of varying scales, MWOA consistently achieves superior performance compared to the other algorithms. The proposed algorithm demonstrates high adaptability in addressing diverse ship replenishment path planning problems, delivering efficient, high-quality, and reliable solutions.

## 1. Introduction

The complex and varied task environment increases the need for maritime resupply when ships conduct missions such as long-distance transportation, patrolling, and humanitarian aid, among others. Ship replenishment path planning [1,2,3] is a crucial task for ensuring the support of maritime operations. Its core objective is to reasonably plan the routes between ships and replenishment points, ensuring the safety of replenishment while completing the replenishment tasks in the shortest time possible. This process needs to consider many uncertain factors like weather, sea conditions, and enemy situations, and the cost of the route. It is very important for improving the efficiency of maritime operations and ensuring the continuous operation of ships.

Based on the practical requirements of real-world maritime operations, this paper formulates a common single-task ship replenishment path planning problem through simplification. The single-task ship replenishment path planning can be understood as the need for ships to go to different replenishment points for supplies before executing tasks. Each replenishment point provides a key item, and the ship needs to traverse all the replenishment points to complete the replenishment. The primary objective is to determine the most efficient route that enables the ship to visit each replenishment point exactly once and return to the starting point. With an increase in replenishment points, the number of potential route combinations grows exponentially, which is a typical NP-hard problem [4]. The path planning challenge examined in this study resembles the traveling salesman problems (TSP) [5,6], and can be approached using both exact algorithms [7] and metaheuristic algorithms [8]. Exact algorithms, including the A* algorithm [9], Dijkstra’s algorithm [10], and dynamic programming [11], ensure the discovery of the optimal solution for small-scale issues. However, their application is constrained by computational limitations when dealing with larger-scale problems. On the other hand, metaheuristic algorithms are preferred for their effectiveness and adaptability in handling large-scale problems. While they may not always yield the optimal solution, they can identify near-optimal solutions within a feasible timeframe, making them particularly suitable for large-scale scenarios; for example, genetic algorithm (GA) [12], hybrid particle swarm optimization (HPSO) [13], ant colony optimization (ACO) [14], simulated annealing (SA) [15], etc. These algorithms do not depend on gradient information and are capable of managing intricate optimization challenges in nonlinear, multi-peak, and high-dimensional environments, showcasing distinct benefits in path planning applications.

At the same time, inspired by nature and physics, a large number of new bio-inspired algorithms [16] have been proposed to solve optimization problems, and these algorithms have great potential in solving path planning problems. These include the sparrow search algorithm [17], the crowned porcupine optimization algorithm [18], the Harris hawks optimization algorithm [19], the hippopotamus optimization algorithm [20], the gray wolf optimizer [21], the symbiotic organisms search [22], etc. Although these algorithms have performed excellently in improving the quality of solutions and global search capabilities, these algorithms are also susceptible to becoming trapped in local optima. To overcome this issue, researchers have proposed a variety of hybrid algorithms and local optimization strategies [23,24,25]. For example, the shuffled frog leaping algorithm [26] combines the advantages of swarm intelligence and local search, optimizing the quality of solutions by simulating the foraging behavior of frogs. Another example is the simulated annealing-based symbiotic organisms search algorithm [27], which merges the simulated annealing algorithm with the symbiotic organisms search algorithm. This combination leverages the global search potential of simulated annealing and the local search efficiency of symbiotic organisms search, significantly boosting the algorithm’s ability to avoid local optima. Additionally, the hybrid discrete artificial bee colony algorithm [28] enhances the artificial bee colony algorithm’s performance in tackling complex optimization problems through discretization and hybrid strategies. Furthermore, Steffen [29] proposed a simplex-enhanced PSO algorithm that strategically repositions particles using Nelder-Mead reflection to escape local optima. This method not only improves PSO’s global search capability but also demonstrates adaptability to other swarm intelligence algorithms. The proposal of these hybrid algorithms and local optimization strategies not only enriches the theoretical system of optimization algorithms but also provides more effective tools for solving complex optimization problems in practical engineering. Through continuous exploration and improvement of these methods, it is expected that greater breakthroughs will be achieved in the research of optimization problems in the future.

Among these bio-inspired algorithms, this paper investigates the innovative application of the whale optimization algorithm (WOA) [30] in the path planning of ship replenishment. As an emerging bionic intelligent optimization algorithm, WOA has attracted much attention for its unique search mechanism and excellent global search capability. The WOA is inspired by the behavioral strategies of whales during foraging and defense. The adaptability and resource optimization allocation capability demonstrated by whales in the natural environment provide new ideas for solving optimization problems. The WOA constructs an efficient search framework by simulating the social behavior and environmental interaction of whales. Each individual in the algorithm represents a potential solution. By simulating the interactions between whales and their environmental adaptability, the algorithm can effectively explore and exploit the global search space to find the optimal solution. Although the whale algorithm has shown great potential, it has not yet been applied to the problem of ship replenishment path planning in practical applications.

The basic WOA tends to encounter issues such as reduced convergence speed and decreased population diversity in the later stages of iteration, making it prone to falling into local optima [31]. To address these challenges, a modified whale optimization algorithm (MWOA) is proposed to more effectively tackle the problem of ship replenishment path planning. The algorithm initially employs a combination of the nearest neighbor search and random search to initialize the population. Subsequently, it introduces crossover operators and a destroy–repair strategy to update the positions of the whales, aiming to enhance the algorithm’s global search capability. Additionally, a variable neighborhood search is employed for local optimization to enhance the quality of the solutions, facilitating the swift and precise planning of optimal replenishment routes in intricate scenarios.

The primary contributions of this research are outlined below:

- A novel algorithm for ship replenishment path planning, termed MWOA, is introduced.
- The proposed MWOA integrates crossover algorithms and destroy–repair operators to achieve a balance between exploration and exploitation capabilities.
- Results and analyses indicate that this approach offers superior competitiveness and robustness in addressing ship replenishment path planning challenges.

The structure of this paper is as follows: Section 2 outlines the model for the ship replenishment planning problem; Section 3 offers an overview of the fundamental WOA and a comprehensive explanation of the proposed MWOA, along with a comparison of MWOA against other established intelligent optimization algorithms to validate its optimization performance and solution quality using standard test sets; Section 4 demonstrates the application of the MWOA to ship replenishment path planning scenarios of varying scales, highlighting its effectiveness and practical utility; finally, Section 5 concludes the study and outlines potential directions for future research.

## 2. Model for the Single-Task Ship Replenishment Path Planning Problem

The single-task ship replenishment path planning problem refers to the rational planning of the routes for ships to various replenishment points on the basis of ensuring the safety of replenishment, with the aim of completing the replenishment operations in the shortest time and returning to the starting point smoothly. However, during the replenishment voyage, ships will encounter many threats, which makes the replenishment task planning complex and challenging. Therefore, it is necessary to incorporate various dangerous threat factors faced by ships into the replenishment task planning to enhance the scientific nature and feasibility of the planning. In view of this, this paper introduces a risk factor *R_i_* into the path planning process and integrates it into the route distance in subsequent path planning. Based on the probability *P_i_* of each replenishment point encountering danger, the risk factor is defined as follows:

(1) $$R_{i}=1+P_{i}$$

To facilitate the study of the ship replenishment path planning, reasonable assumptions are made regarding certain conditions of the problem:

(1) There are no obstacles between the replenishment points, allowing passage along the shortest path in a Euclidean plane;
(2) All replenishment vessels and equipment operate stably, maintaining the same navigation speed and replenishment speed at all times;
(3) Assuming the starting point is replenishment point 1 (*s* = 1).

To facilitate modeling, the concepts of the shortest distance and the shortest risk distance are introduced. The shortest distance refers to the distance of the entire route without considering the weight of the risk areas, and its formula is as follows:

(2) $$\min Z=\sum_{i=1}^{n}\sum_{j=1,j\neq i}^{n}d_{ij}x_{ij}$$

where *d_ij_* represents the distance between replenishment points *i* and *j*, *n* is the number of replenishment points, and each replenishment point *i* provides a specific type of material replenishment, *x_ij_* is a binary decision variable, which is equal to 1 if the ship travels directly from replenishment point *i* to replenishment point *j*, and 0 otherwise.

The shortest risk distance refers to the distance that a ship travels through risk areas, taking into account not only the straight-line distance of the route but also the comprehensive impact of the risk areas on the entire route. To reduce computational complexity, we avoid explicit risk factor assignment to individual edges. The risk factor for the path between points *i* and *j* is modeled as *R_i_* × *R_j_*, where *R_i_* is the risk factor of replenishment point *i*, and *R_j_* is the risk factor of replenishment point *j*. The specific formula of shortest risk distance is as follows:

(3) $$\min Z=\sum_{i=1}^{n}\sum_{j=1,j\neq i}^{n}d_{ij}x_{ij}R_{i}R_{j}$$

Based on the above model assumptions, the mathematical model for ship replenishment path planning is as follows:

(4) $$\min Z=\sum_{i=1}^{n}\sum_{j=1,j\neq i}^{n}d_{ij}x_{ij}R_{i}R_{j}$$

(5) $$s.t.\;\;\;\;\;\;\;\;\;\;\sum_{j=1,j\neq i}^{n}x_{ij}=1,\;\forall i\in v$$

(6) $$\sum_{i=1,i\neq j}^{n}x_{ij}=1,\;\forall j\in v$$

(7) $$\sum_{i,j\in S,i\neq j}x_{ij}\leq |S|-1,\;\forall S\subseteq v,2\leq |S|\leq n-1$$

(8) $$x_{ij}\in \{0,1\},\;\forall i,j\in v,i\neq j$$

where *v* represents the set of replenishment points, *v* = {1, 2,…, *n*}, *S* is the number of nodes contained in the sub-ring when some nodes are selected from the set *v* of all nodes to form a sub-ring.

## 3. Modified Whale Optimization Algorithm

### 3.1. WOA [30]

The WOA is inspired by the hunting behavior of humpback whales, specifically their use of the “bubble-net feeding” technique to capture prey. The algorithm aims to find the optimal solution by mimicking the self-organization and adaptability of whale groups. The WOA primarily involves three key phases: encircling prey, bubble-net feeding, and searching for prey. In the WOA, each whale’s position corresponds to a potential solution, and by iteratively updating these positions within the solution space, the algorithm converges to the global optimum.

The primary steps of the WOA are as follows:

(1) Initialization: Generate a random set of whale positions and velocities to initialize the algorithm. These positions represent potential solutions, referred to as whale individuals, which are candidates for the optimal solution in the solution space. Additionally, configure the algorithm’s parameters, such as the number of whales and the maximum number of iterations.
(2) Fitness Evaluation: Determine the fitness score for each whale, evaluating the quality of its solution based on the objective function of the optimization problem. Whales with higher fitness scores indicate better solutions.
(3) Encircling Prey: Assume that the optimal individual in the current population is the prey (i.e., the current optimal solution) and the other whale individuals in the population encircle the position of the optimal whale (or a randomly selected whale) to update their own positions. This process is achieved through specific mathematical formulas, enabling the whale individuals to gradually approach the optimal solution. The formula for updating the position is as follows:

(9) $$X\left(t+1\right)=X^{*}\left(t\right)-A\times D$$

(10) A=2×a×rand−a

(11) $$D=\left|\begin{array}{c} C\times X^{*}\left(t\right)-X\left(t\right) \end{array}\right|$$

(12) C=2×rand

where *t* is the current iteration number, *A* is the encircling coefficient, *D* is the distance between the whale and the prey, *C* is a random coefficient, *X* denotes the position of the current solution, X* is the position of the current optimal solution, *a* is a linearly decreasing weight that gradually reduces from 2 to 0 during the iteration process, and *rand* is a random vector in the range from 0 to 1, inclusive.

(4) Bubble-net feeding: This simulates the spiral movement of whales around their prey, applying this process to local search. Individual whales gradually approach the prey (the current optimal solution) through a spiral ascent. This step is also achieved through specific mathematical formulas, including two sub-steps of encircling and spiral updating. The formula for updating the position is as follows:

(13) $$X(t+1)=D^{*}\times e^{bl}\times \cos(2\pi l)+X^{*}(t)$$

where D* represents the absolute value of the distance between the whale and the prey, *b* and *l* are parameters controlling the shape of the spiral, and *e* is the base of the natural logarithm.

(5) Random Search: In some cases, individual whales perform random searches to enhance exploration capabilities and avoid becoming trapped in local optima. When specific conditions are met (such as |*A*| being greater than or equal to 1), whales randomly search and prey based on their positions. The position update formula used for encircling prey is applied.
(6) Update global best solution: The optimal global solution is updated based on fitness information to guide the next search step of the whale individuals.
(7) Iteration: The aforementioned steps are repeated until the stopping conditions are met.

### 3.2. Nearest Neighbor Search

The nearest neighbor search [32,33] is a simple and intuitive method for initializing candidate solutions. This algorithm starts from a random replenishment point and successively selects the nearest unvisited replenishment point as the next node to visit, thereby generating a relatively optimal initial path. However, the nearest neighbor method primarily relies on known good solutions to generate the initial population, which may lead to individuals in the population being overly similar and lacking diversity. To enhance the quality and diversity of the initial population, MWOA employs a combination of nearest neighbor search and random search for population initialization. The nearest neighbor method generates high-fitness individuals based on known good solutions, rapidly elevating the overall level of the initial population; random search introduces diverse individuals, expanding the search space and preventing the population from falling into local optima. The synergistic effect of these two approaches not only fully utilizes existing information but also strengthens the algorithm’s global search capability, achieving a balance between exploration and exploitation, thereby improving optimization efficiency and the quality of solutions.

### 3.3. Crossover Operation

In GA, the crossover operation effectively enhances population diversity by recombining parental genes to generate new offspring, helping the algorithm escape local optima and explore a broader search space. At the same time, it preserves and combines excellent gene segments, allowing offspring fitness to increase rapidly and accelerating algorithm convergence. Moreover, the crossover operation balances exploration and exploitation, expanding new solutions while utilizing excellent genes to enhance algorithm performance, making it a key component in solving complex optimization problems. Based on this, this paper applies the cyclic crossover [34] and subtour exchange crossover [35] from genetic algorithms to MWOA.

**Figure 1:**
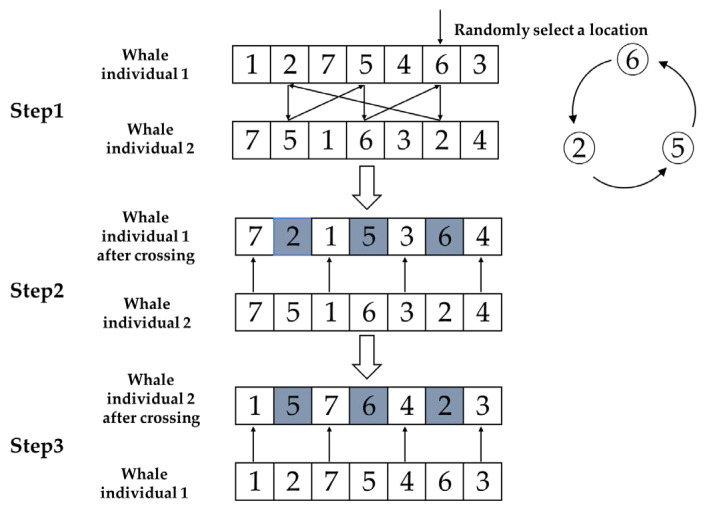
Schematic diagram of cyclic crossover.

Cyclic Crossover: Randomly selects one gene from a parent, then finds the gene number at the corresponding position on another parent, returning to the first parent to locate the position of the same-numbered gene, and the previous steps are repeated until a loop is formed. The positions of all genes in the loop are the final selected positions. Generate offspring using the selected genes from parent chromosome 1, ensuring positional correspondence, and finally place the remaining genes from parent chromosome 2 into the offspring. The other offspring is obtained in the same manner, as shown in Figure 1. The characteristic of cyclic crossover is that only one position needs to be randomly selected to obtain multiple crossover positions.

**Figure 2:**
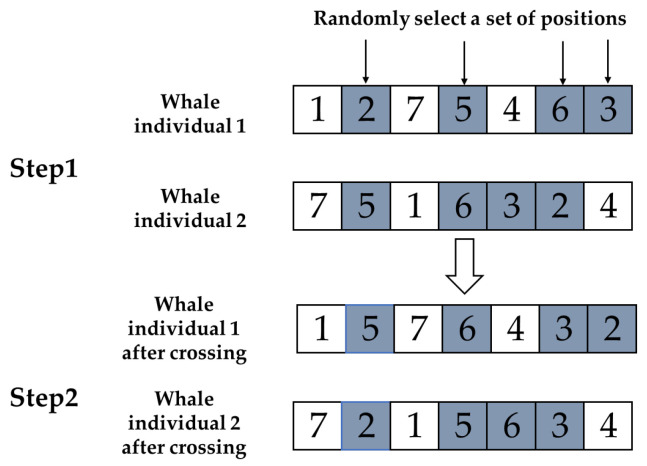
Schematic diagram of subtour exchange crossover.

Subtour exchange crossover: A group of genes is selected from one parent and these genes are located on another parent. Keep the unselected genes unchanged and, according to the order of appearance of the selected genes, exchange the positions of genes in the chromosomes of the two parents, generating two offspring at once. The specific operation process is shown in Figure 2.

### 3.4. Destroy and Repair Operators

The destroy and repair operators are heuristic algorithm operators commonly used in the solution of combinatorial optimization problems, especially widely applied in fields such as vehicle routing problems and scheduling problems. In this paper, the Destroy Operator employs Random Destruction [36], which involves randomly selecting a subset of elements to remove from the current solution. The Repair Operator utilizes Regret Repair [37,38], considering multiple potential insertion positions and selecting the one that can maximize the improvement of the objective function for insertion.

### 3.5. Variable Neighborhood Search (VNS)

The VNS [39] is an improved local search algorithm. The fundamental idea is to systematically change the set of neighborhood structures during the search process to expand the search, obtain a local optimal solution, and then, based on this local optimal solution, systematically change the set of neighborhood structures again to expand the search range and find another local optimal solution. For path optimization problems, VNS can optimize routes through alternating searches of different neighborhood structures.

The neighborhood structure set chosen in this paper includes swap, reverse, and insert operations. The swap neighborhood involves exchanging the positions of two cities, the reverse neighborhood involves selecting a subtour and reversing its order, and the insert neighborhood involves inserting a city into another position. Regarding the current solution and the optimal solution in the optimization process, perturb the current path according to the neighborhood strategy (swap, reverse, insert) to generate a new candidate solution. If the new path obtained through neighborhood search is shorter than the currently recorded optimal path, update the optimal path and the length of the optimal path. Variable Neighborhood Search, through this iterative perturbation and neighborhood search, can conduct a broader exploration in the solution space, thereby helping to find a more optimal solution.

### 3.6. MWOA

For the problem of ship replenishment path planning, this paper makes appropriate modifications to the WOA. In the initialization phase, a strategy combining the nearest neighbor search with random search is adopted for population initialization. Regarding the update strategy of whale individuals, cyclic crossover is used to replace the original search and foraging phase, subtour exchange crossover is used to replace the original spiral update strategy, and the destroy and repair operators are used to replace the original encircling phase. Additionally, variable neighborhood search is employed for local search to improve the quality of the solution.

The pseudocode for the MWOA is presented in Algorithm 1.
**Algorithm 1: Modified whale optimization algorithm**1.Initialize the number of whales *N* and the maximum number of iterations *T*, etc.2.Perform population initialization *X_i_* (*i* = 1, 2,…, *N*) using a combination of nearest neighbor search and random search.3.**While** *t* < *T* **do**4.Check if any whale has exceeded the search space and make modifications accordingly.5.Calculate the fitness value for each whale and identify the whale that is the most satiated X*6.**For** *i* = 1 **to** *N* **do**7.**Update *A***8.**If** rand < 0.5 **then**9.**If** |*A*| < 1 **then**10.Execute the destroy and repair operators.11.**Else**12.Execute cyclic crossover.13.**End If**14.**Else**15.Execute subtour crossover.16.**End If**17.Execute variable neighborhood search.18.**End For**19.*t* = *t* + 120.**End While**21.Return the optimal solution X*

### 3.7. Testing the Effectiveness of MWOA

The ship replenishment problem studied in this paper is similar to the TSP problem. To test the effectiveness of the improved algorithm, six test datasets were selected from the standard TSPLIB dataset (available at http://comopt.ifi.uni-heidelberg.de/software/TSPLIB95/ (accessed on 10 October 2024)) for testing the improved algorithm, namely ulysses22, eil51, st70, kroA100, ch130, and tsp225. The TSP problem was solved using WOA, GA, HPSO, ACO, and SA methods, and the results were compared and analyzed with MWOA. To guarantee precision and consistency, and to minimize the influence of occasional results on the evaluation of algorithm performance, each algorithm was executed 30 times. For all algorithms, the population size was fixed at 50, with a maximum of 500 iterations.

**Figure 3:**
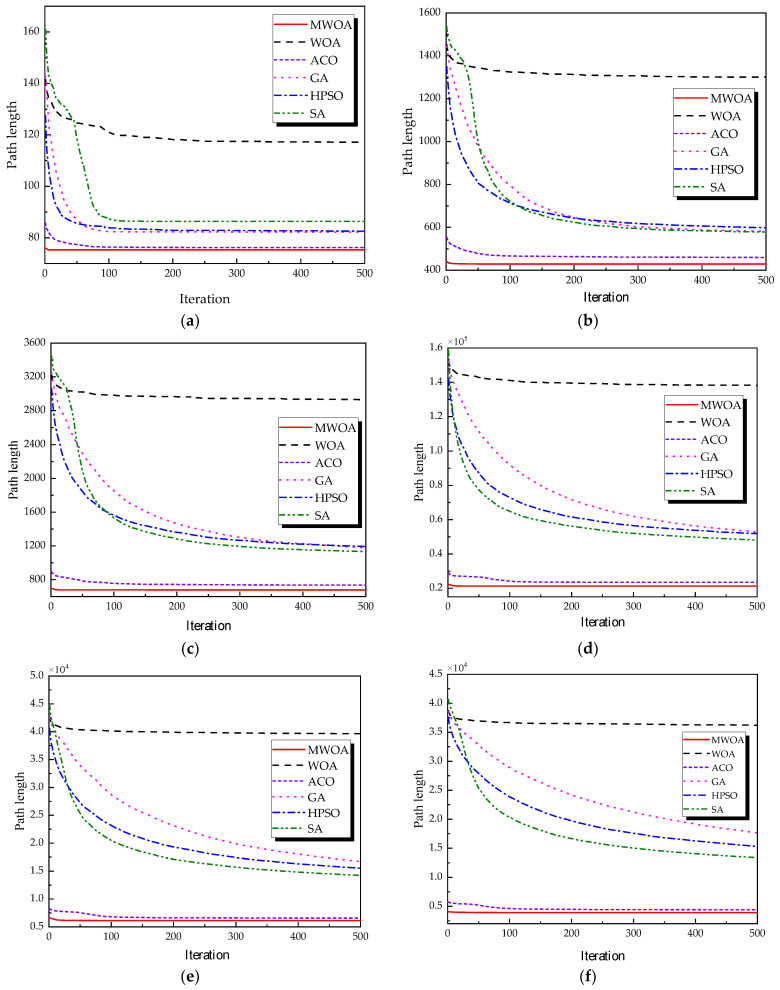
The average convergence curves derived from 30 runs of different algorithms. (**a**) Ulysses22; (**b**) eil51; (**c**) st70; (**d**) kroA100; (**e**) ch130; and (**f**) tsp225.

**Figure 4:**
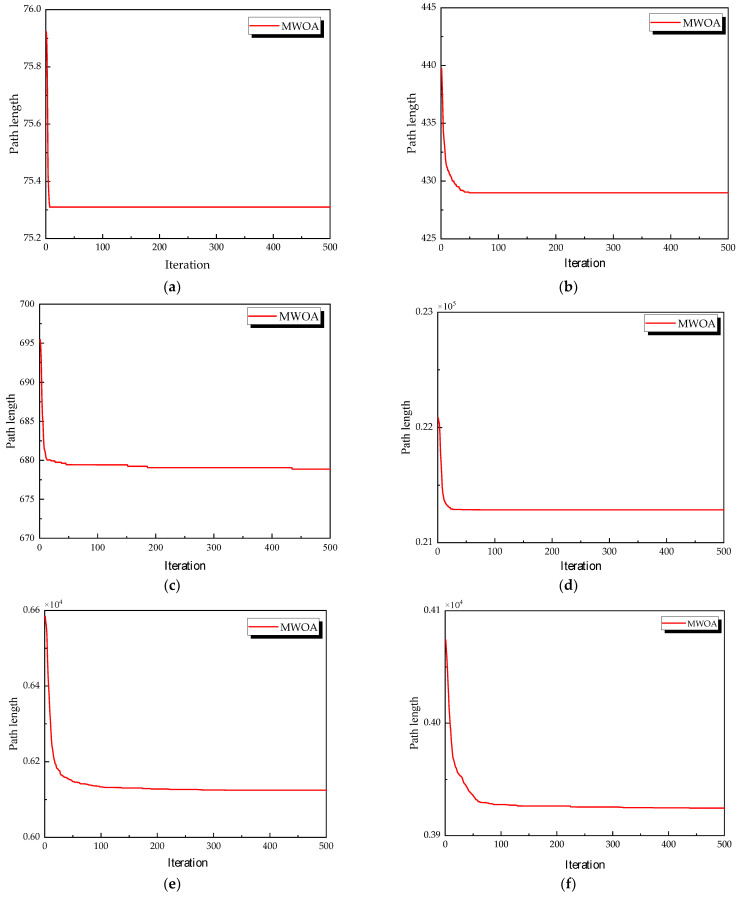
The average convergence curves derived from 30 runs of MWOA. (**a**) Ulysses22; (**b**) eil51; (**c**) st70; (**d**) kroA100; (**e**) ch130; and (**f**) tsp225.

**Figure 5:**
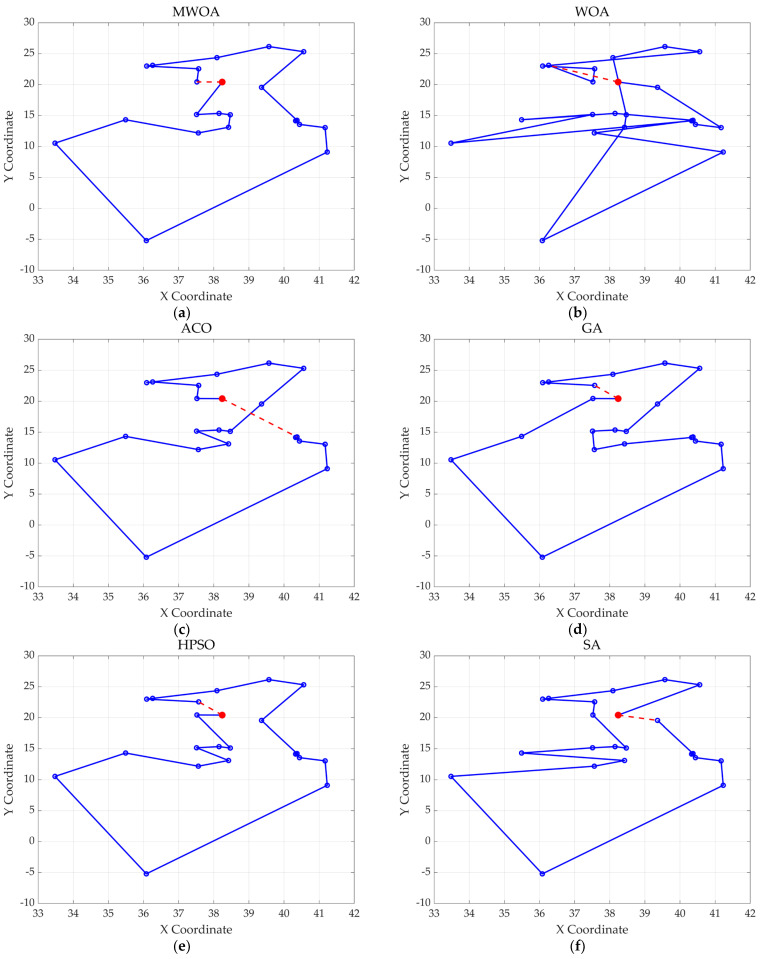
The optimal paths of six algorithms in the ulysses22 case. (**a**) MWOA; (**b**) WOA; (**c**) ACO; (**d**) GA; (**e**) HPSO; and (**f**) SA.

**Figure 6:**
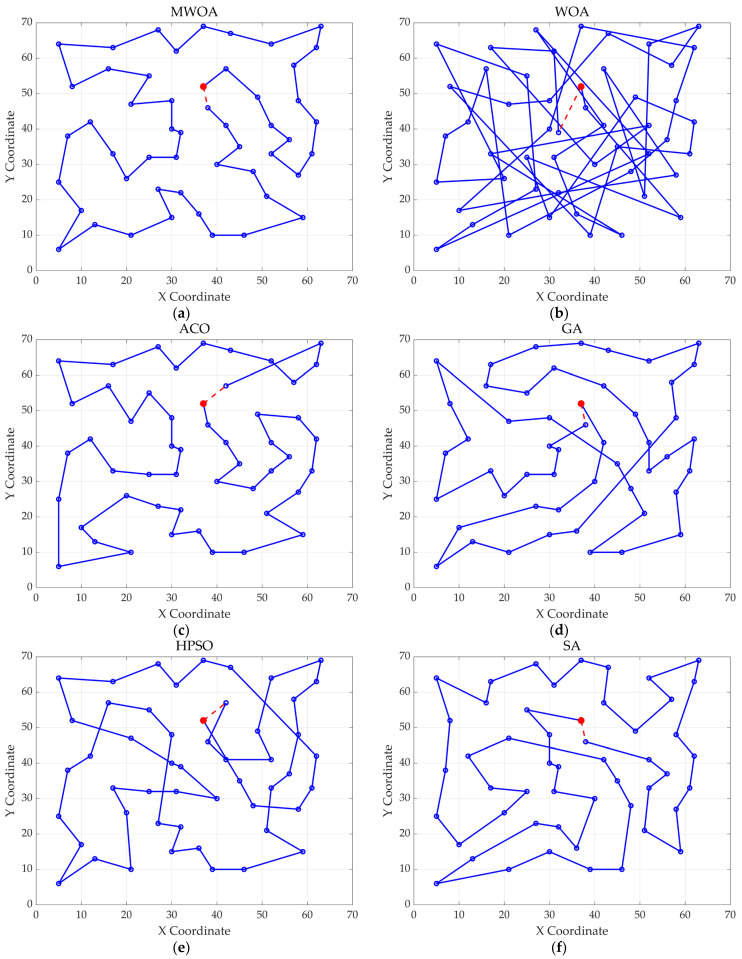
The optimal paths of six algorithms in the eil51. (**a**) MWOA; (**b**) WOA; (**c**) ACO; (**d**) GA; (**e**) HPSO; and (**f**) SA.

**Figure 7:**
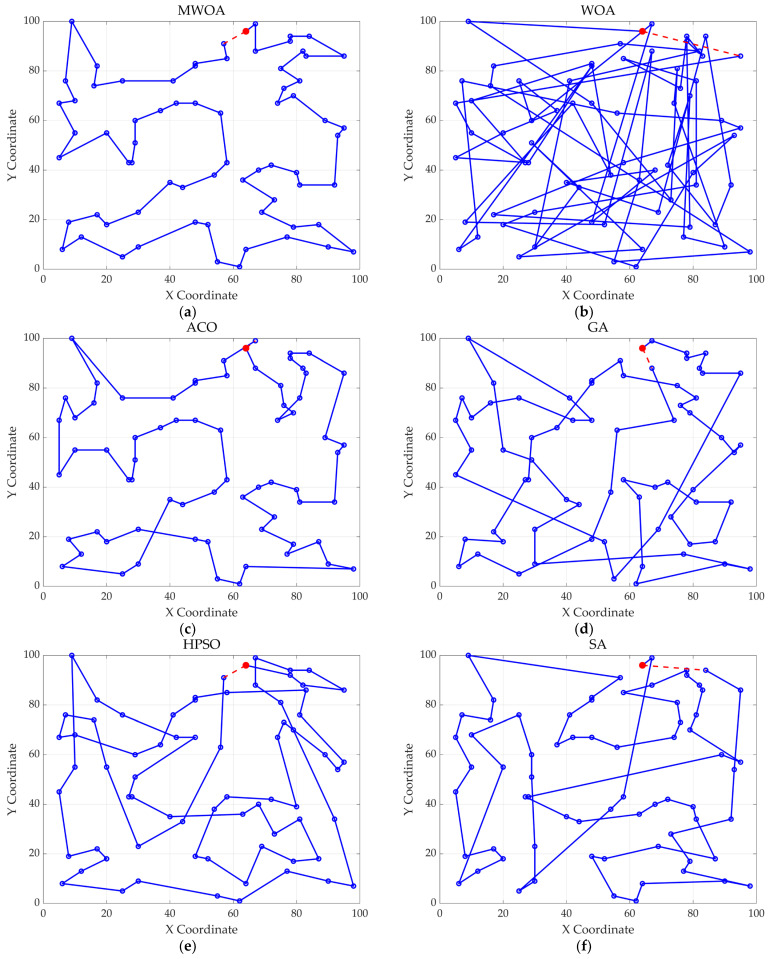
The optimal paths of six algorithms in the st70. (**a**) MWOA; (**b**) WOA; (**c**) ACO; (**d**) GA; (**e**) HPSO; and (**f**) SA.

**Figure 8:**
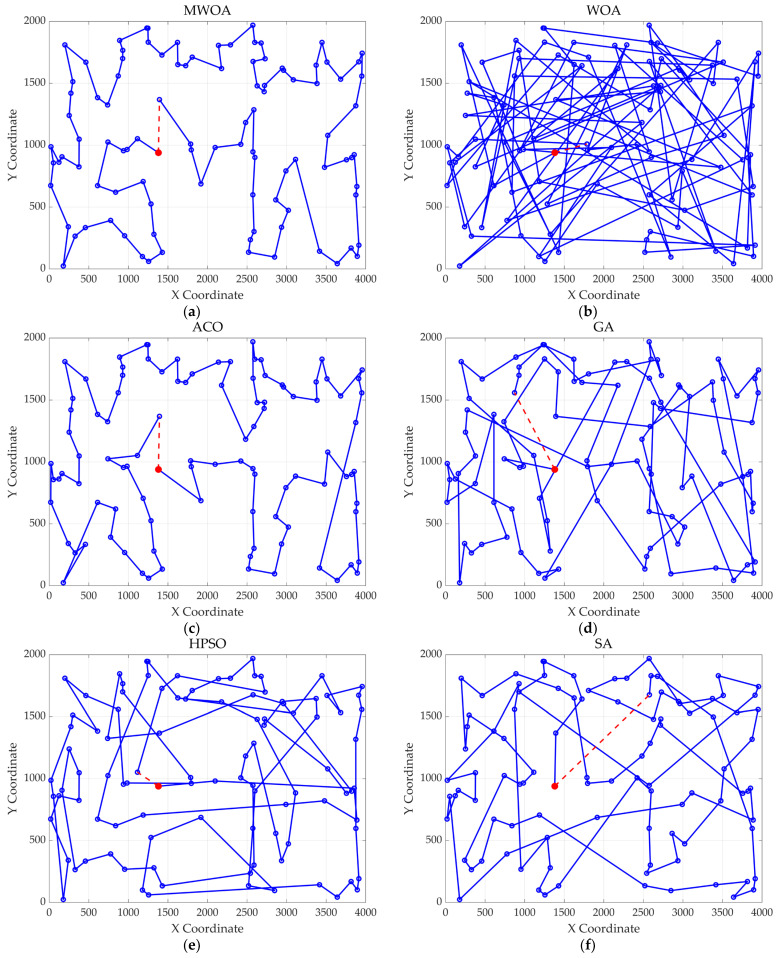
The optimal paths of six algorithms in the kroA100 case. (**a**) MWOA; (**b**) WOA; (**c**) ACO; (**d**) GA; (**e**) HPSO; and (**f**) SA.

**Figure 9:**
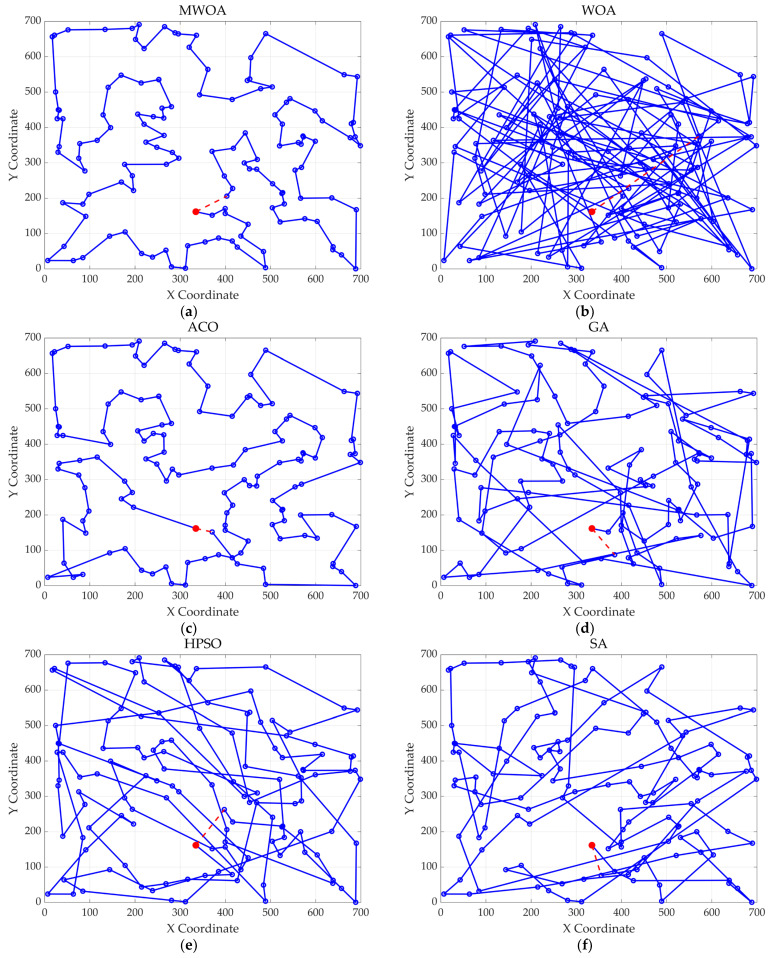
The optimal paths of six algorithms in the ch130 case. (**a**) MWOA; (**b**) WOA; (**c**) ACO; (**d**) GA; (**e**) HPSO; and (**f**) SA.

**Figure 10:**
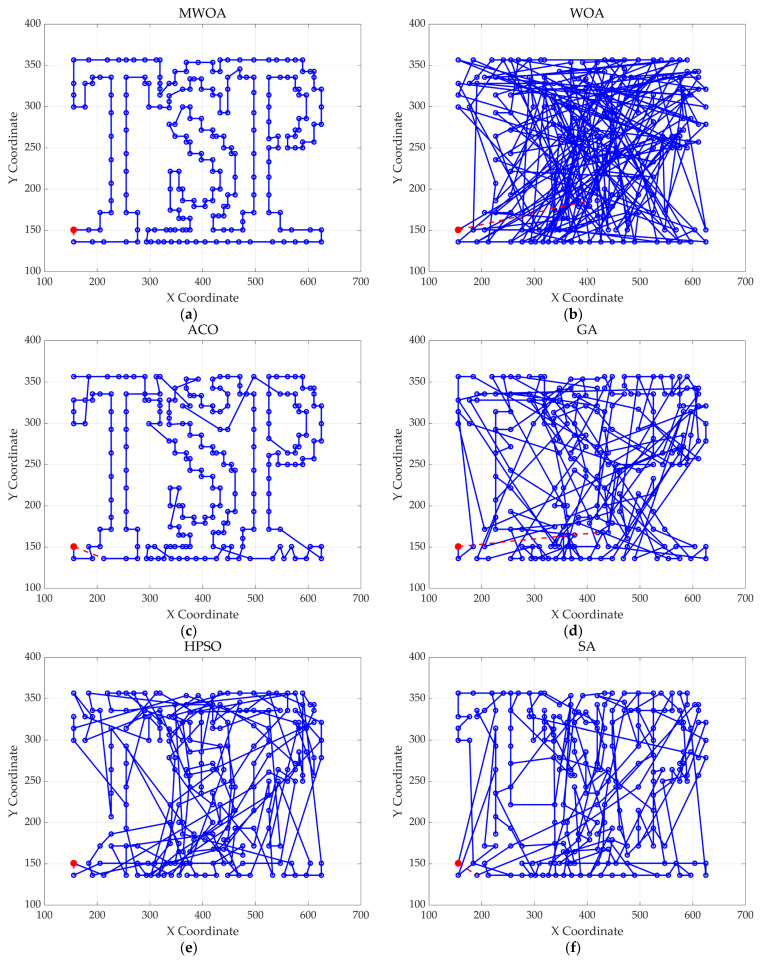
The optimal paths of 6 algorithms in the stp225 case. (**a**) MWOA; (**b**) WOA; (**c**) ACO; (**d**) GA; (**e**) HPSO; and (**f**) SA.

From each run, the best solution (minimum objective value) was recorded. The following metrics were calculated across all 30 runs: “Best” representing the best solution among all 30 runs, “Mean” reflecting the average of the 30 best solutions, “Std” quantifying the standard deviation of the 30 best solutions. These metrics were used to compare the performance of the MWOA, WOA, GA, HPSO, ACO, and SA algorithms, as summarized in Table 1. Figure 3 shows the average convergence curves derived from 30 independent runs of the MWOA, WOA, GA, HPSO, ACO, and SA algorithms, where each run generates a trajectory of the best fitness values across iterations. Figure 4 shows the average convergence curves derived from 30 independent runs of the MWOA separately for clearer performance visualization. Figure 5, Figure 6, Figure 7, Figure 8, Figure 9 and Figure 10 show the optimal path diagrams corresponding to the “Best” metric of the MWOA, WOA, GA, HPSO, ACO, and SA algorithms for each test dataset.

Taking ulysses22 as an example for analysis, the results shown in Figure 3a indicate that even when taking the average of 30 runs, the MWOA demonstrates a more pronounced convergence rate and optimal fitness value compared to other functions. The reason for this is that the MWOA uses a combination of the nearest neighbor search and random search to generate the initial population. This strategy allows it to achieve far superior optimal results in the first generation, thereby effectively promoting the rapid convergence of the entire algorithm. The average value, best value, and standard deviation of the improved algorithm listed in Table 1 are also superior to other algorithms, and the best value and average value of the MWOA are quite close with a small error, indicating that the improved algorithm has good stability and is not easily affected by initial conditions or random factors, possessing strong adaptability and anti-interference capability. The optimal path diagrams listed in Figure 5, Figure 6, Figure 7, Figure 8, Figure 9 and Figure 10 also intuitively demonstrate the advantages of this algorithm.

Furthermore, for test cases such as eil51, st70, kroA100, ch130, and tsp225, the improved algorithm also shows excellent performance. This not only verifies the universality of the MWOA in handling TSP-like path planning problems but also highlights its superior performance in solving such problems, providing efficient, stable, and reliable solutions for path planning tasks of various scales and complexities.

## 4. Application of MWOA to Single-Task Ship Replenishment Path Planning

**Figure 11:**
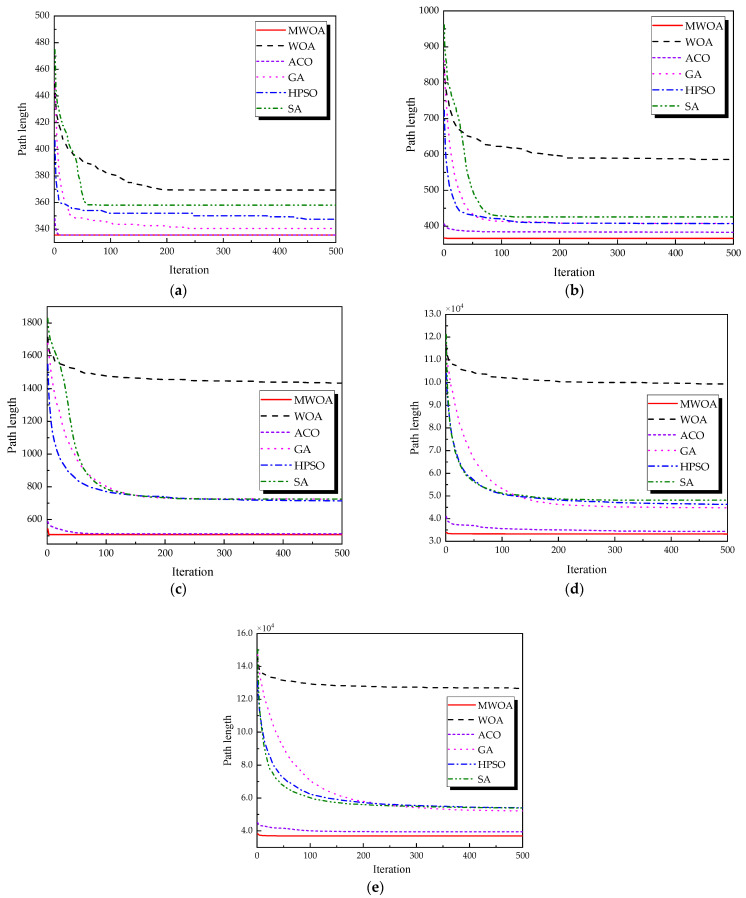
The average convergence curves derived from 30 runs of different algorithms. (**a**) 11 replenishment points; (**b**) 20 replenishment points; (**c**) 32 replenishment points; (**d**) 39 replenishment points; and (**e**) 48 replenishment points.

**Figure 12:**
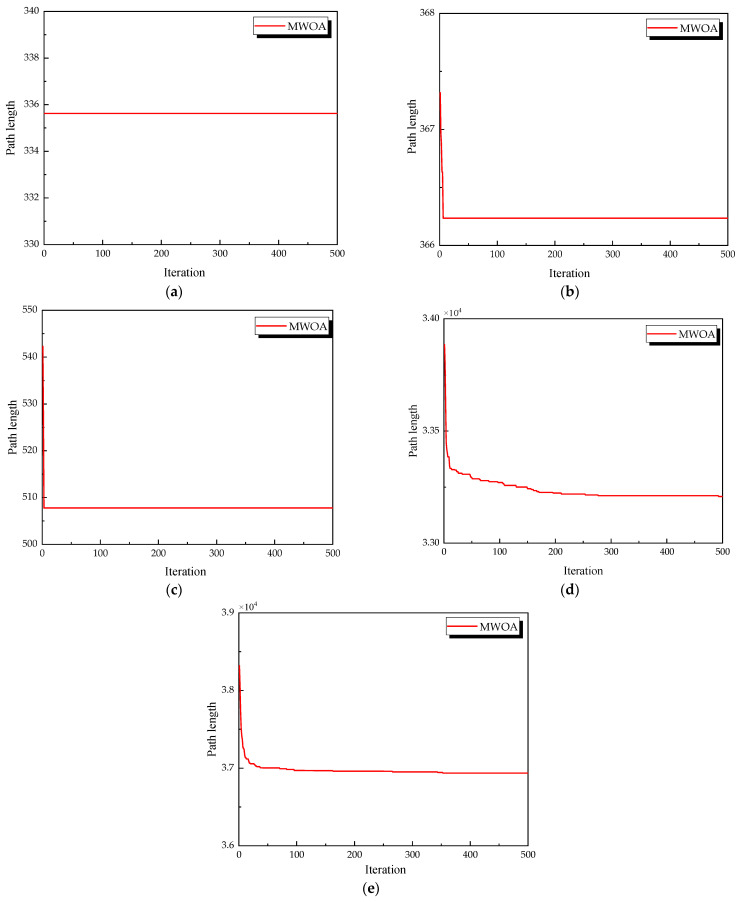
The average convergence curves derived from 30 runs of MWOA. (**a**) 11 replenishment points; (**b**) 20 replenishment points; (**c**) 32 replenishment points; (**d**) 39 replenishment points; and (**e**) 48 replenishment points.

**Figure 13:**
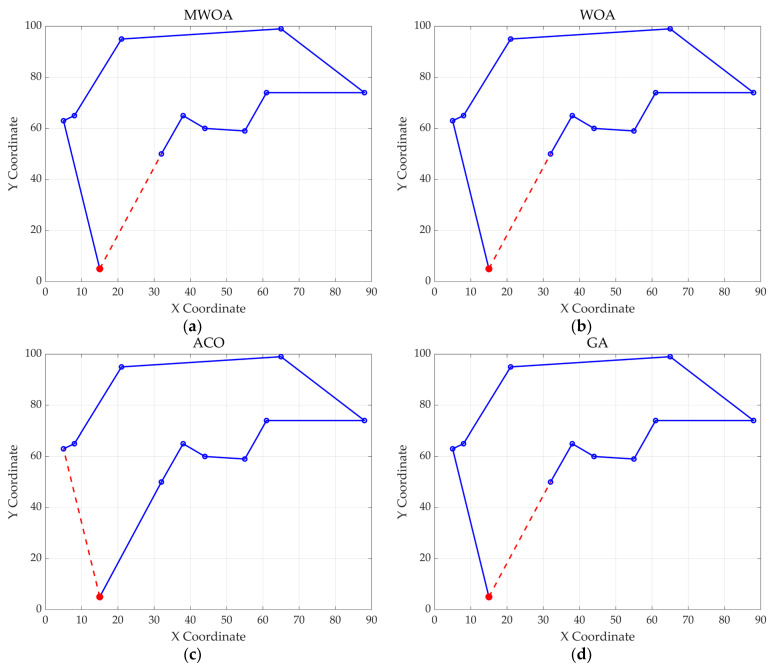

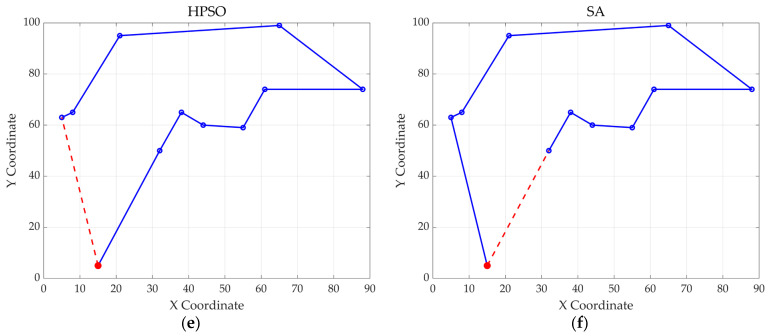
The optimal paths of six algorithms in 11 replenishment point cases. (**a**) MWOA; (**b**) WOA; (**c**) ACO; (**d**) GA; (**e**) HPSO; and (**f**) SA.

**Figure 14:**
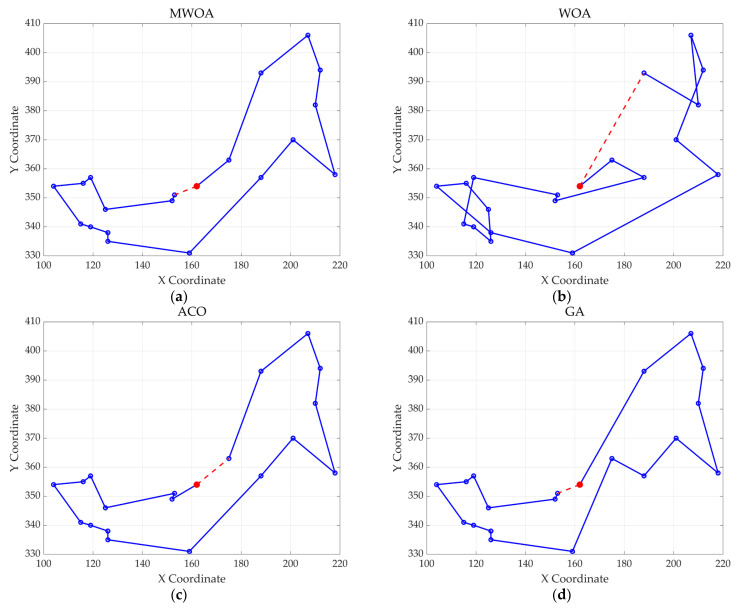

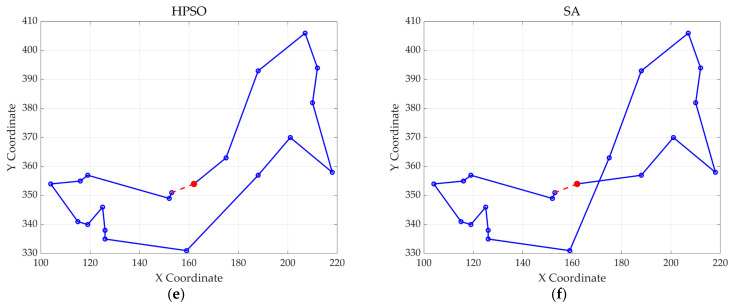
The optimal paths of six algorithms in 20 replenishment point cases. (**a**) MWOA; (**b**) WOA; (**c**) ACO; (**d**) GA; (**e**) HPSO; and (**f**) SA.

**Figure 15:**
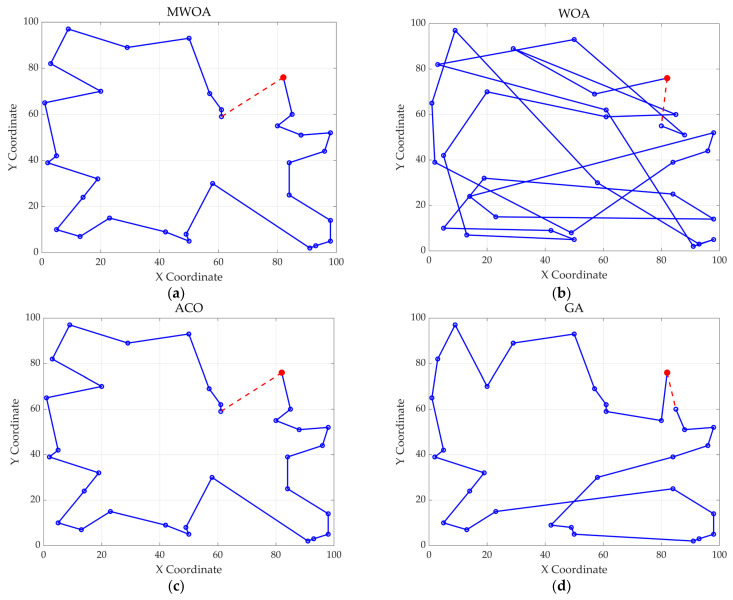

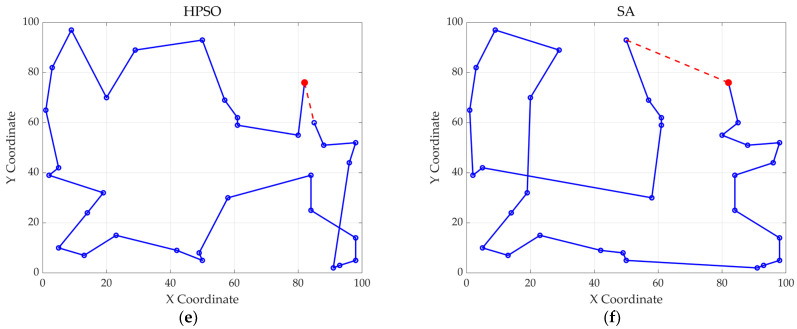
The optimal paths of six algorithms in 32 replenishment point cases. (**a**) MWOA; (**b**) WOA; (**c**) ACO; (**d**) GA; (**e**) HPSO; and (**f**) SA.

**Figure 16:**
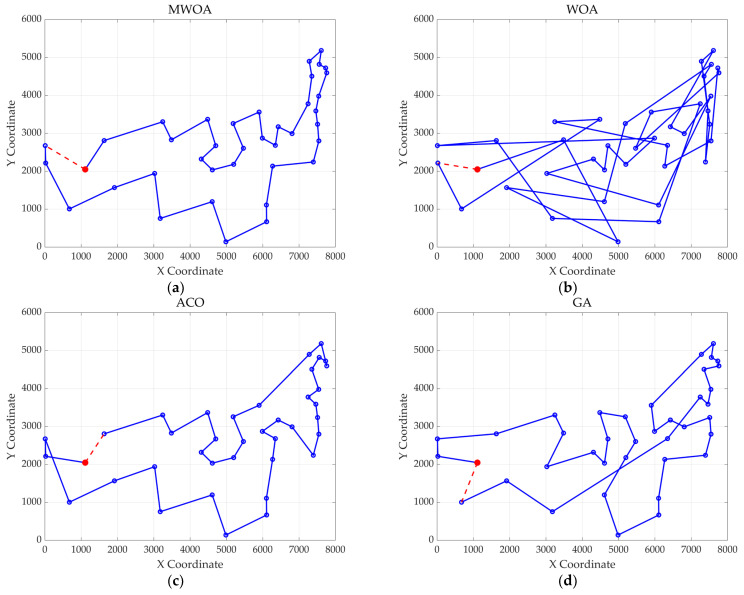

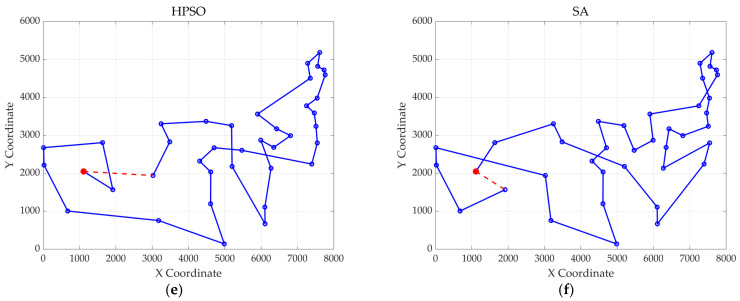
The optimal paths of six algorithms in 39 replenishment point cases. (**a**) MWOA; (**b**) WOA; (**c**) ACO; (**d**) GA; (**e**) HPSO; and (**f**) SA.

**Figure 17:**
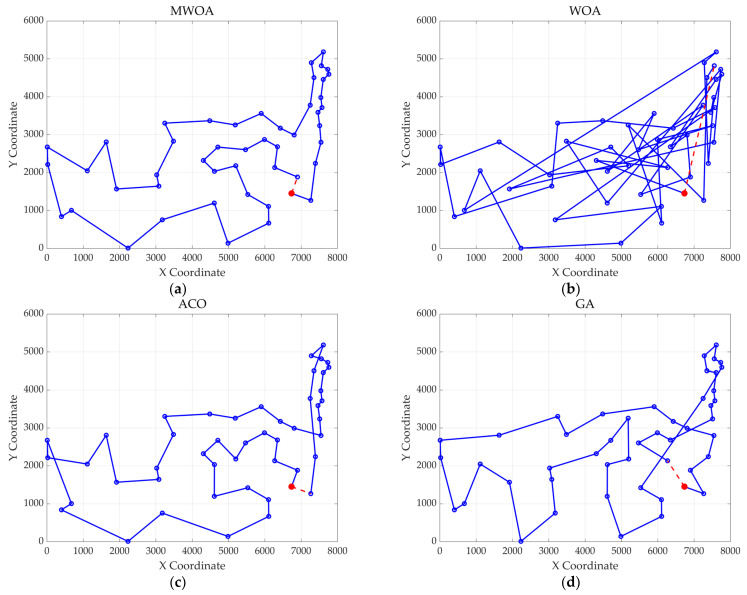

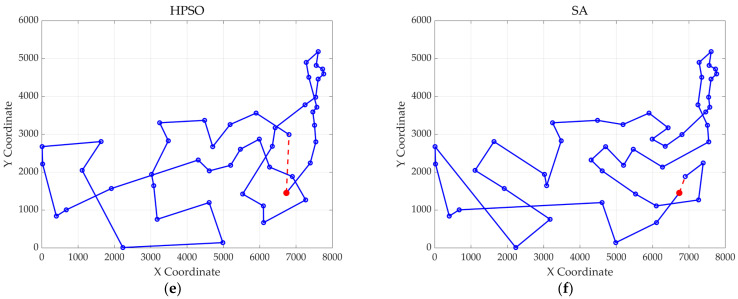
The optimal paths of six algorithms in 48 replenishment point cases. (**a**) MWOA; (**b**) WOA; (**c**) ACO; (**d**) GA; (**e**) HPSO; and (**f**) SA.

This chapter applies the MWOA to solve the single-task ship replenishment path planning problem, discussing five cases as examples, including 11, 20, 32, 39, and 48 replenishment points, respectively. The specific coordinates of the replenishment points and the probabilities of encountering danger are randomly assigned. The probability of risk occurrence ranges from 0 to 0.2. The WOA, GA, HPSO, ACO, and SA algorithms were used to solve these five problems, and the results were compared and analyzed with the MWOA. For all algorithms, the population size was fixed at 50, and the maximum number of iterations was capped at 500. Each algorithm was executed 30 times, and the best values, average values, and standard deviation of the results from 30 independent runs were compared and analyzed. Figure 11 shows the average convergence curves derived from 30 independent runs of the MWOA, WOA, GA, HPSO, ACO, and SA algorithms. Figure 12 shows the average convergence curves derived from 30 independent runs of the MWOA separately for clearer performance visualization. Figure 13, Figure 14, Figure 15, Figure 16 and Figure 17 show the optimal path diagrams corresponding to the “Best” metric of the MWOA, WOA, GA, HPSO, ACO, and SA algorithms for each test dataset. Table 2 lists the statistical data of the best values, average values, and standard deviation of all algorithms after 30 runs for the five cases.

As shown in Figure 11, even when taking the average of 30 runs, the MWOA still demonstrates a significantly faster convergence rate and optimal fitness value compared to other algorithms. The initialization of the population using a combination of the nearest neighbor search and random search allows the MWOA to achieve far superior optimal results in the first generation, thereby accelerating the entire convergence process of the algorithm. In the context of ship replenishment path planning, a good initial population provides a starting point that is closer to the optimal solution for the subsequent optimization process, enabling the algorithm to more quickly select the best path from numerous possible route options.

As shown in Table 2, the improved algorithm outperforms other algorithms in key indicators such as average value, best value, and standard deviation. Moreover, the best value of MWOA is almost the same as the average value, and the error is also at a low level. This series of data fully proves that the improved algorithm has excellent stability and can effectively resist the interference of initial conditions or random factors. The stability of the supply path planning scheme is extremely critical in ship supply missions. A stable planning result can ensure that the ship efficiently and safely completes its supply tasks according to the established plan, avoiding additional risks caused by the uncertainty of the planning scheme, such as supply delays, navigation safety issues, etc., thereby ensuring the stable operation of the entire ship supply system.

The optimal path diagrams presented in Figure 13, Figure 14, Figure 15, Figure 16 and Figure 17 intuitively highlight the many advantages of this algorithm, strongly confirming its excellent performance in solving ship replenishment path planning problems. In summary, the MWOA shows high adaptability in ship replenishment path planning. Its fast convergence, strong stability, strong adaptability, and high-quality planning results make it capable of providing efficient, reliable, and high-quality path planning solutions for ship replenishment tasks, with broad application prospects and significant practical value.

## 5. Conclusions

This study innovatively proposes an enhanced version of the whale optimization algorithm specifically designed to address the problem of ship supply path planning. The study first used a mixed method of the nearest neighbor search and random search for population initialization. Subsequently, the population is updated using crossover operations and destroy and repair operators, thereby substantially improving the global search efficiency of the whale optimization algorithm. Furthermore, the algorithm incorporates variable neighborhood search for local optimization, thereby enhancing its overall search performance.

To thoroughly assess the efficacy of the proposed algorithm, this research initially evaluated its optimization capabilities using six classic TSPs. The results indicate that, compared to other algorithms, the MWOA demonstrates superior performance in both solving ability and stability. Ultimately, the MWOA was applied to ship replenishment path planning of different scales, and the results showed that the algorithm has significant advantages in this field compared to the comparative algorithms, demonstrating strong practical application potential and promising to provide efficient and accurate path planning solutions for ship replenishment tasks.

However, this study still has some limitations. The current research work mainly focuses on the optimization of the single-ship replenishment route length and has not yet fully considered more complex and practical factors such as multi-ship replenishment and capacity constraints. Future research can explore the application performance of the MWOA in multi-ship replenishment with capacity constraints, in order to expand the application boundaries of the algorithm, enabling it to more accurately and comprehensively serve the actual ship replenishment path planning problems and provide stronger theoretical and technical support for the efficient execution of ship replenishment tasks.

## Figures and Tables

**Table 1:** Performance comparison of different algorithms in six typical TSP.

Function		MWOA	WOA	ACO	GA	HPSO	SA
ulysses22	Best	75.31	102.90	75.97	75.91	75.76	76.12
	Ave	75.31	117.18	76.20	82.18	82.57	86.37
	Std	4.26 × 10^−14^	5.88	0.08	5.47	5.98	5.76
eil51	Best	428.98	1234.91	447.66	530.17	526.56	516.07
	Ave	428.98	1300.60	459.24	582.60	598.70	578.26
	Std	2.84 × 10^−13^	27.28	3.87	28.64	40.45	33.90
st70	Best	677.11	2817.60	715.23	1040.53	1003.47	1022.78
	Ave	678.86	2930.75	735.21	1178.41	1193.80	1134.27
	Std	2.70	54.33	10.01	81.70	103.03	51.81
kroA100	Best	21,285.44	132,944.30	22,483.69	44,994.97	45,050.49	41,217.24
	Ave	21,285.44	138,097.00	23,465.66	52,827.86	51,920.57	48,071.72
	Std	1.09 × 10^−11^	2189.19	294.91	4035.75	3919.93	3418.13
ch130	Best	6110.72	38,581.12	6439.33	14,623.08	14,294.17	12,983.13
	Ave	6124.76	39,663.67	6577.05	16,717.00	15,533.87	14,237.33
	Std	24.86	406.58	54.01	766.11	721.97	643.24
tsp225	Best	3894.53	35,022.90	4289.05	15,741.32	13,957.01	12,251.55
	Ave	3924.48	36,198.54	4387.65	17,619.23	15,301.83	13,341.45
	Std	10.08	469.08	45.02	651.38	609.20	546.82

**Table 2:** Performance comparison of different algorithms in five ship replenishment cases.

Function		MWOA	WOA	ACO	GA	HPSO	SA
11 replenishment points	Best	335.63	339.05	335.63	335.63	335.63	335.63
	Ave	335.63	369.34	335.63	340.55	347.53	358.07
	Std	5.78 × 10^−14^	17.44	5.78 × 10^−14^	10.23	13.27	17.07
20 replenishment points	Best	366.23	498.23	369.69	375.41	369.01	377.08
	Ave	366.23	586.25	383.12	407.87	407.21	425.74
	Std	5.78 × 10^−14^	57.18	4.05	30.60	31.35	46.68
32 replenishment points	Best	507.78	1336.05	507.78	583.67	535.57	574.90
	Ave	507.78	1434.03	512.49	723.44	714.02	725.00
	Std	2.31 × 10^−13^	40.96	3.49	66.08	79.13	70.14
39 replenishment points	Best	33,195.76	84,134.97	33,988.03	39,214.31	38,527.20	39,450.69
	Ave	33,207.94	99,360.14	34,359.24	44,779.90	46,323.33	48,151.56
	Std	37.35	4799.32	255.68	3913.70	5071.25	4238.67
48 replenishment points	Best	36,808.78	117,039.90	38,332.55	42,168.43	44,666.96	45,000.74
	Ave	36,936.84	126,649.08	39,439.21	52,239.47	54,061.49	53,948.61
	Std	110.68	3987.72	421.53	5641.83	4417.54	4183.19

## Data Availability

The data presented in this study are available on request from the corresponding author.
